# Recognition of Linear B-Cell Epitope of Betanodavirus Coat Protein by RG-M18 Neutralizing mAB Inhibits Giant Grouper Nervous Necrosis Virus (GGNNV) Infection

**DOI:** 10.1371/journal.pone.0126121

**Published:** 2015-05-04

**Authors:** Chien-Wen Chen, Ming-Shan Wu, Yi-Jen Huang, Chao-An Cheng, Chi-Yao Chang

**Affiliations:** 1 Institute of Cellular and Organismic Biology, Academia Sinica, Taipei, Taiwan; 2 Institute of Fisheries Science, National Taiwan University, Taipei, Taiwan; 3 Department of Food Science, National Quemoy University, Kinmen, Taiwan; CEA, FRANCE

## Abstract

Betanodavirus is a causative agent of viral nervous necrosis syndrome in many important aquaculture marine fish larvae, resulting in high global mortality. The coat protein of Betanodavirus is the sole structural protein, and it can assemble the virion particle by itself. In this study, we used a high-titer neutralizing mAB, RG-M18, to identify the linear B-cell epitope on the viral coat protein. By mapping a series of recombinant proteins generated using the *E*. *coli* PET expression system, we demonstrated that the linear epitope recognized by RG-M18 is located at the C-terminus of the coat protein, between amino acid residues 195 and 338. To define the minimal epitope region, a set of overlapping peptides were synthesized and evaluated for RG-M18 binding. Such analysis identified the _195_VNVSVLCR_202_ motif as the minimal epitope. Comparative analysis of Alanine scanning mutagenesis with dot-blotting and ELISA revealed that Valine_197_, Valine_199_, and Cysteine_201_ are critical for antibody binding. Substitution of Leucine_200_ in the RGNNV, BFNNV, and TPNNV genotypes with Methionine_200_ (thereby simulating the SJNNV genotype) did not affect binding affinity, implying that RG-M18 can recognize all genotypes of Betanodaviruses. In competition experiments, synthetic multiple antigen peptides of this epitope dramatically suppressed giant grouper nervous necrosis virus (GGNNV) propagation in grouper brain cells. The data provide new insights into the protective mechanism of this neutralizing mAB, with broader implications for Betanodavirus vaccinology and antiviral peptide drug development.

## Introduction

Viral nervous necrosis (VNN) [1], otherwise known as viral encephalopathy and retinopathy (VER) [2], is an infectious neuropathological disease that has spread to more than 40 species of marine and freshwater fish worldwide, and results in nearly 100% mortality in infected larvae and juvenile stage fish [3, 4]. Typical clinical symptoms include abnormal swimming behavior, such as darting and spiral swimming; in addition, vacuolating necrosis can be observed in components of the central nervous system, such as brain, retina, and spinal cord. The disease causative agents are members of the genus *Betanodavirus* of the family *Nodaviridae*, also known as nervous necrosis viruses (NNV). Betanodavirus has a small (about 25 nm) non-enveloped icosahedral structure containing a bipartite, linear, single-strand, positive-sense RNA genome composed of RNA1 (3.1 Kb) and RNA2 (1.4 Kb), which encode a RNA-dependent RNA polymerase and a coat protein, respectively [3, 4]. In addition to RNA1 and RNA2, a subgenomic transcript of RNA1, RNA3 (0.4 Kb), encodes protein B2; this protein is a suppressor involved in post-transcriptional gene silencing, and it antagonizes host cell RNA interference mechanisms during virus multiplication [5, 6]. Based on the similarity of the T4 variable region of RNA2 sequences encoding the C-terminal halves of the coat protein, four genotype strains have been phylogenetically classified: redspotted grouper nervous necrosis virus (RGNNV), striped jack nervous necrosis virus (SJNNV), barfin flounder nervous necrosis virus (BFNNV), and tiger puffer nervous necrosis virus (TPNNV) [7]. More recently, a Betanodavirus isolated from turbot, *Scophthalmus maximus* (TNV), was suggested to belong to a fifth genotype [8, 9]. The RNA2 nucleotide sequence identities among these five genotypes are 76 to 82%, and those of coat protein amino acid sequences are 77 to 86% [9]. In spite of the sequence similarities, these five genotypes exhibit different host tropism: RGNNV has a broad host range, causing diseases in a variety of fish species, especially groupers and sea bass, whereas BFNNV has only been isolated from some coldwater species [3, 8–10]. SJNNV and TPNNV have been isolated from clinically-infected striped jack *Pseudocaranx dentex* and tiger puffer *Takifugu rubripes* in Japan, respectively. However, SJNNV and SJNNV/RGNNV reassortant were also found in symptomatic fish in Europe including European sea bass *Dicentrarchus labrax*, sea bream *Sparus aurata*, and Senegalese sole *Solea senegalensis* [3, 9, 11–13]. Several factors influence host specificity for viral multiplication, including environmental adaptability (e.g. temperature), host defense systems (e.g. immune systems and posttranscriptional gene silencing), and host intracellular factors, as well as target cell receptors for viral entry. Binding between such receptors and Betanodavirus coat protein is important for host specificity determination. *In vivo* experiments showed that the host specificity of SGNNV (a member of the RGNNV type) and SJNNV to their original host fish larvae, sevenband grouper and striped jack, respectively, is controlled by the T4 variable region of viral RNA2 and/or encoded coat protein via reassortant chimeric viruses from SJNNV and SGNNV [14, 15].

As the sole capsid protein, Betanodavirus coat protein is required for viral particle assembly, viral diagnostic recognition, virus entry signaling, and host specificity. Several prophylaxes have been developed to control diseases caused by Betanodavirus, including neutralizing antibodies, which can be used for both viral diagnosis [16, 17] and antibody-based therapies [18]. Neutralizing antibodies protect the host from viral infection via binding directly to virus particles, which leads to inhibition of virus attachment to host cells or interference with viral entry processes, like penetration, uncoating, or subsequent transcription [19]. Mapping of epitopes through peptide scanning can be used to characterize the immunodominant antigen of the virion surface bound by the neutralizing antibody. The comprehensive analysis of neutralizing epitopes will facilitate the design of effective peptide drugs against viral infection. However, relatively few epitopes on the Betanodavirus capsid protein have been identified. Previously, Nishizawa et al. [20] identified a putative 3-mer B-cell epitope located at amino acid residues 254–256 of the coat protein, based on binding patterns of differential mABs against SJNNV recombinant proteins expressed from *Escherichia coli* [20]. Costa et al. [21] subjected serum samples from naturally infected sea bass and neutralizing mABs to Pepscan to identify a 32-mer region of the Betanodavirus capsid protein spanning amino acid residues 181–212 [21]. In the present study, the epitope of the RGNNV genotype coat protein was mapped by Pepscan using RG-M18 [16], a high-titer (6.5 log_10_ Neutralization index) neutralizing mouse IgG1 isotype monoclonal antibody against yellow grouper (*Epinephelus awoara*) nervous necrosis virus (YGNNV) [22], a member of the RGNNV genotype. The neutralizing epitope was identified as an 8-mer linear amino acid region, and the crucial amino acid residues of the epitope were determined by the Alanine-scanning method. Furthermore, the 8-mer epitope was found to present in a Lysine-scaffold octamer form, which exhibited propagation inhibitory activity against giant grouper (*Epinephelus lanceolatus*) nervous necrosis virus (GGNNV) infection.

## Materials and Methods

### Viruses and cells

The GB3 cell line [23] was cultured in Leibovitz’s L-15 media supplemented with 10% heat-inactivated fetal bovine serum (FBS) (Gibco, USA), L-glutamine (Gibco, USA), and penicillin-streptomycin (Gibco, USA) at 28°C. The yellow grouper nervous necrosis virus (YGNNV) [22] and giant grouper nervous necrosis virus (GGNNV) of the RGNNV genotype were propagated from the tissue culture supernatant of virus infected-GB3 cells, as described previously [23]. Briefly, aliquots of 10^5^ GB3 cells ml^-1^ were seeded into each well of a 24-well tissue culture plate (BD FALCON, USA) and incubated at 28°C until a monolayer with a confluence of 80% formed. The monolayer of cells was washed with PBS, and a series of 10-fold serial dilutions of virus stock in L-15 media containing 2% FBS was used to inoculate each well. After incubation at 28°C for 7 days, the cytopathic effect (CPE) was observed with an inverted microscope (Axiovert 200M Zeiss/Photometrics CoolSnap HQ, Germany), and the titer was calculated by the TCID_50_ method described by Reed & Muench [24]. Of the wells with CPE, the well inoculated with the greatest dilution was selected, and its supernatant was passed through a 0.22 μm filter (Merck Millipore, Germany) and collected in a sterile tube. The cultured virus was stored at 4°C and used for the following experiments within 2 weeks.

### Preparation of monoclonal antibody

The RG-M18 mouse hybridoma cell line was established in 2001 [16]. To prepare RG-M18 mAB, hybridoma cells were cultured in Hybird-SFM (Gibco, USA) media in 175T flasks at 37°C with 5% CO_2_ supplement. Every 3 to 5 days, the supernatant was collected when the media turned yellow. Antibody was purified using Protein G Agarose Fast Flow (Merck Millipore, Germany), following the manufacturer’s instructions: supernatant was loaded onto the Protein G packaged column, and the column was then washed with a volume of ice-cold PBS equal to 10-fold that of the column. The bound antibody was eluted with 50 mM glycine (Merck, Germany) pH 2.7 (1 ml per fraction in tubes containing 10 × neutralization buffer (1 M Tris (Merck, Germany) pH 8, 1.5 M NaCl (Merck, Germany), 1 mM EDTA(Merck, Germany)). The purified antibody was stored in 50% glycerol (Merck, Germany) and 0.03% NaN_3_ (Merck, Germany) at -20°C.

### Construction of plasmids

Plasmid pET20b-1A59, which encodes full-length (1014 bp) YGNNV coat protein, was used as a template to amplify DNA fragments encoding serial deletions of coat protein. Primers designed for construction of serial coat protein deletions are listed in Table 1. Each PCR was carried out using a mixture of 1 ng template DNA, 0.2 μM forward primer, 0.2 μM reverse primer, 0.2 mM of each dNTP, 5 μl 10 × *Pfu* buffer, 2 mM MgSO_4_, and 2.5 U *Pfu* DNA Polymerase (Fermentas, USA) in a final volume of 50 μl. PCR amplification was performed in an automatic thermal cycler (MyCycler thermal cycler, BioRad, USA) with the following program: one cycle at 94°C for 5 min, followed by 35 cycles of 94°C for 30 s, 63°C for 30 s, and 72°C for 60 s, and finally one cycle at 72°C for 7 min. DNA fragments were extracted using a QIAquick PCR Purification kit (Qiagen, Germany) in accordance with the manufacturer’s instructions. The purified DNA fragments were digested with *Nde*I and *Xho*I (New England Biolabs, UK), and ligated into pET-20b(+) (Merck Novagen, USA) plasmids cleaved by the same enzymes. The pET-20b(+) vector provides a sequence encoding His-tag, enabling the generation of a His-tag fusion protein that can be purified through immobilized metal affinity chromatography.

**Table 1:** Primers for construction of recombinant YGNNV coat protein plasmids.

Primer	Sequence
NNVCP*Nde*I	GGAATTCCATATGGTACGCAAAGGTGAGAAGAAATTGG
NNVCP*Nde*I151F	GGAATTCCATATGGGGACCAATGACGTCCATCTCTC
NNVCP*Nde*I583F	GGAATTCCATATGGTCAACGTGTCAGTACTGTGTC
NNVCP300*Xho*I	CCGCTCGAGCTGGAAGATTCTAGCAGCGTG
NNVCP450*Xho*I	CCGCTGGAGGGCAACGACTGCACCGCG
NNVCP600*Xho*I	CCGCTCGAGCAGTACTGACACGTTGACCAC
NNVCP*Xho*I	CCGCTCGAGGTTTCCCGAGTCAACCCTGGTG

### Expression and purification of recombinant proteins

After sequence confirmation, pET-20b(+) plasmids with varying lengths of YGNNV coat protein gene were used to transform the BL-21 (DE3) strain of *Escherichia coli* (Merck Novagen, USA). Transformed *E*.*coli* BL-21 was cultured in 100 ml LB broth with 100 μg ampicillin (AMRESCO, USA) ml^-1^ at 37°C with shaking (150 r.p.m.). When the OD_600_ reached about 0.4 to 0.6, the liquid culture was cooled to 25°C, and IPTG (MDBio Inc., Taiwan) was added to a final concentration of 0.4 mM to induce expression of recombinant protein, and incubated for 4 h with shaking at 200 r.p.m. Cells were harvested and suspended in 10 ml binding buffer (20 mM sodium phosphate (Merck, Germany), 0.5 M NaCl, 20 mM imidazole (Merck, Germany), 8 M urea (SIGMA-ALDRICH, USA), pH 7.4), and sonicated using a Digital Sonifier (Branson, USA). The supernatant of the lysate was passed through a column with 1 ml His60 Ni Superflow resin (Clontech, USA) equilibrated with 10 ml binding buffer. After washing with 10 ml binding buffer, the recombinant protein was eluted by the addition of 10 ml elution buffer (20 mM sodium phosphate, 0.5 M NaCl, 500 mM imidazole, 8 M urea, pH 7.4), 1 ml per fraction in each tube.

### SDS-PAGE and Western blotting analyses

SDS-PAGE and Western blotting of serial deletion constructs were performed to map the epitopes of recombinant coat protein recognized by RG-M18. Purified recombinant proteins from the deletion clones were separated by 18% SDS-PAGE and transferred onto a PVDF membrane (Immobilon-P, Merck Millipore, Germany) with an ECL semi-dry blotter (Amersham, UK). The PVDF membrane was blocked with 5% (w/v) skimmed milk in 0.05% (v/v) TWEEN 20 (J.T. Baker, USA)/TBS (TBST) at 37°C for 30 min. After incubation with 1:1,000 diluted RG-M18 mAB in 5% skimmed milk at 37°C for 1 h, the membrane was washed twice with TBST buffer (15 min/wash). Secondary antibody hybridization was performed with polyclonal goat anti-mouse immunoglobulin/AP (D0486, Dako, USA) at a dilution of 1:5,000 in TBST buffer at 37°C for 1 h. After two 15 min washes with TBST buffer, the PVDF membrane was developed under BCIP/NBT substrate solution (PerkinElmer, USA) in the dark. Development was stopped by washing with ddH_2_O, and the images were captured using a UVP BioSpectrum 600 Image System (UVP Inc., USA). To determine the efficiency at which the synthetic epitope peptide inhibited viral propagation, Western blot was used to examine Betanodavirus coat protein amount in GGNNV infected-GB3 cells incubated in the presence of different concentrations of 8-mer multiple antigen peptide (MAP) (see next section for details). The GB3 cell monolayer was incubated in a 10-cm tissue culture dish (BD FALCON, USA) with L-15 media containing 2% FBS and final concentrations of 0, 5, 10, 20, or 40 μg ml^-1^ MAP or mutant 8-mer MAP (m-MAP) (the latter served as a negative control; see next section for details). After 1 h incubation, each well was inoculated with GGNNV at an m.o.i. = 10 in L-15 media containing 2% FBS. Following a further 1 h incubation period, cells were washed with PBS and then incubated in L-15 media containing 2% FBS for 24 h. Total proteins were collected from infected-GB3 cell debris, and separated by 15% SDS-PAGE. The same procedure and antibodies for Western blot analysis were used as described above. Anti-actin antibody (MAB1501, Merck Millipore, Germany) at a dilution of 1:10,000 was used to detect actin protein as an internal control. The intensities of Betanodavirus coat protein and actin bands were quantified using VisionWorksLS Analysis Software Ver 6.8 of the UVP BioSpectrum 600 Image System.

### Peptide synthesis and dot-blotting analyses

To further refine the epitope region determined using serial deletion recombinant proteins of YGNNV coat protein, synthetic peptides were used for dot-blotting analyses. Peptides were synthesized using the CEM Liberty automated microwave peptide synthesizer (Matthews, NC, USA) with Wang resin (for single peptide synthesis) or Fmoc-8-branch MAPS resin (for multiple antigen peptide); synthesis was performed at the Core Facility of the Institute of Cellular and Organismic Biology, Academia Sinica. Purified peptides was determined using the microflex LRF MALDI-TOF system (Bruker Daltonics, Inc., USA). Each synthetic 20-mer peptide overlapped with its predecessor by 10 residues; these peptides corresponded to the 195–338 aa region of the YGNNV coat protein, and were used in dot-blotting to evaluate the epitope-binding ability of RG-M18 mAB. Synthetic 8-mer peptides with an overlap of 6 residues (from aa 195 to 206) were further used to narrow down the epitope region by dot-blotting. To explore the contribution of each residue in the _195_VNVSVLCR_202_ epitope, Alanine substitution mutagenesis of synthetic 8-mer peptides was combined with dot-blotting and ELISA analyses. The region corresponding to the epitope in SJNNV _195_VNVSVMCR_202_ has a M200L substitution, and therefore the synthetic peptide _195_VNVSVLCR_202_ was used as control for dot-blotting and ELISA analyses. The 8-mer multiple antigen peptide (MAP) was synthesized based on the sequence, _195_VNVSVLCR_202_, using an 8-branch Lysine scaffold; the ability of MAP to compete with virus infection was examined. The mutant 8-mer multiple antigen peptide (m-MAP), _195_VNASVLAR_202_ with V197A and C201A double mutations, was synthesized for use as a control. For dot-blotting analyses, all synthetic peptides were dissolved in DMSO (SIGMA-ALDRICH, USA) at a concentration of 10 mg ml^-1^. Each 2 μl (20 μg) peptide sample was spotted on a PVDF membrane pre-wetted with methanol (Merck, Germany), and the membrane was air dried before blocking. After blocking with 5% skimmed milk in TBST buffer at 37°C for 30 min, the PVDF membrane was incubated with 1:1,000 diluted RG-M18 mAB in 5% skimmed milk at 37°C for 1 h. The membrane was then washed, and subjected to secondary antibody hybridization with polyclonal goat anti-mouse immunoglobulin/AP at a dilution of 1:5,000 in TBST buffer for 1 h at 37°C. After washing, the PVDF membrane was developed under BCIP/NBT substrate solution in the dark for 15 min, and the images were captured using a UVP BioSpectrum 600 Image System. For dot-blotting, YGNNV recombinant coat protein (virus-like particle, VLP, 1–338 aa) and synthetic peptides 195–214 aa and 195–202 aa were used as positive controls. All dot-blotting analyses were performed in triplicate.

### ELISA

To further confirm the results obtained from dot-blotting, the same synthetic peptides were subjected to ELISA analysis. Each well of a Nunc-ELISA plate (Thermo Scientific, USA) was coated with 100 μl of single peptide (10 mg ml^-1^) dissolved in DMSO, and plates were incubated at 4°C for 15 h. The plates were then rinsed three times with PBS containing 0.05% (v/v) TWEEN 20 (PBST), and then blocked with PBST containing 5% skimmed milk at 37°C for 2 h. After three washes with PBST, 100 μl of a 1:500 dilution of culture media containing RG-M18 mAB was added to each well. After a 2-h incubation at 37°C, plates were rinsed three times with PBST, and subjected to secondary antibody hybridization with a 1:2,500 dilution of polyclonal goat anti-mouse immunoglobulin/AP in PBST buffer at 37°C for 1 h. Following three washes with PBST, enzyme activity was determined by adding 100 μl of chromogenic substrate (pNPP: p-nitrophenyl phosphate, (S0942 SIGMA-ALDRICH,USA)) dissolved in glycine buffer (0.1 M glycine, pH 10.4, with 1 mM MgCl_2_ (Merck, Germany) and 1 mM ZnCl_2_ (Merck, Germany)). After a 30-min incubation in the dark, the OD of the resulting yellow product was read at 405 nm with a microtiter plate reader (SpectraMax M5, Molecular Devices, USA). YGNNV recombinant coat protein (VLP, 1–338 aa) and synthetic peptides 195–214 aa and 195–202 aa were used as positive controls. Seven replicates (n = 7) were performed for statistical analysis.

### Peptide competitive inhibition assay

Viral neutralizing epitopes possess the ability to interfere with viral entry, and therefore inhibit viral multiplication. To address this possibility, an 8-branch Lysine scaffold 8-mer multiple antigen peptide (MAP) _195_VNVSVLCR_202_ was synthesized to perform peptide competitive inhibition assays. For image analysis, GB3 cells were cultured in a 6-well tissue culture plate (BD FALCON, USA) until a monolayer with a confluence of 80% was reached. The monolayer was washed with PBS, and then incubated in L-15 media containing 2% FBS and a final concentration of 0, 5, 10, 20, or 40 μg ml^-1^ MAP. After 1 h incubation, each well was inoculated with GGNNV (m.o.i. = 10), then continuously incubated at 28°C. The CPE of treated cells was recorded by inverted microscopy at 0, 24, 48, and 72 h. For the viral titration assay, GB3 cells were cultured in a 24-well tissue culture plate until a monolayer with a confluence of 80% was reached. The monolayer was washed with PBS, and then incubated in L-15 media containing 2% FBS and a final concentration of 0, 5, 10, 20, or 40 μg ml^-1^ MAP or m-MAP (control). After 1 h incubation, each well was inoculated with a series of 10-fold dilutions of GGNNV (10^7^ TCID_50_ ml^-1^), and then incubated at 28°C. The CPE of treated cells was continuously recorded for 7 days by inverted microscopy and the infective titers were determined by the TCID_50_ method described by Reed and Muench (24). The titration assay was performed in triplicate.

### Immunocytochemical staining

To further detect inhibition of viral propagation by synthetic neutralizing epitope peptide, MAP, immunocytochemical staining with RG-M18 mAB was performed. A GB3 cell monolayer cultured on a 4-well glass Millicell EZ slide (PEZGS0416, Merck Millipore, Germany) was washed with PBS, and then incubated in L-15 media containing 2% FBS and a final concentration of 0, 5, 10, 20, or 40 μg ml^-1^ MAP for 1 h. GGNNV (m.o.i. = 10) in L-15 media containing 2% FBS was then added to each well. Following a 1-h inoculation, cells were washed with PBS, and then incubated in L-15 media containing 2% FBS for 24 h at 28°C. The treated cells were fixed in 4% paraformaldehyde (Merck, Germany) for 15 min at room temperature. After three washes with PBS, cells were permeabilized by incubation with 0.25% Triton X-100 (Merck, Germany) in PBS for 5 min at room temperature. Cells were washed twice more with PBS, and then blocked by incubation with 10% bovine serum albumin in PBS (10% BSA/PBS) for 30 min, before being incubated with RG-M18 mAB in 3% BSA/PBS (1:200 dilution) for 2 h at room temperature. The cells were rinsed three times with PBS, and then incubated with goat anti-mouse antibody-rhodamine (AP124R, Chemicon, USA) in 3% BSA/PBS (1:1000 dilution) for 30 min at room temperature. After rinsing three times with PBS, the cells was incubated with DAPI (Invitrogen, USA) (1:20,000 dilution with PBS, stock: 5 mg ml^-1^ in ddH_2_O) in the dark for 10 min at room temperature. After a further three washes with PBS, the slides were mounted in Fluoromount Aqueous Mounting Medium (SIGMA-ALDRICH, USA) and covered by a coverslip for imaging analysis under fluorescence microscopy (Axiovert 200M Zeiss/Photometrics CoolSnap HQ).

## Results

### Mapping Betanodavirus coat protein epitope by Western blotting

**Fig 1:**
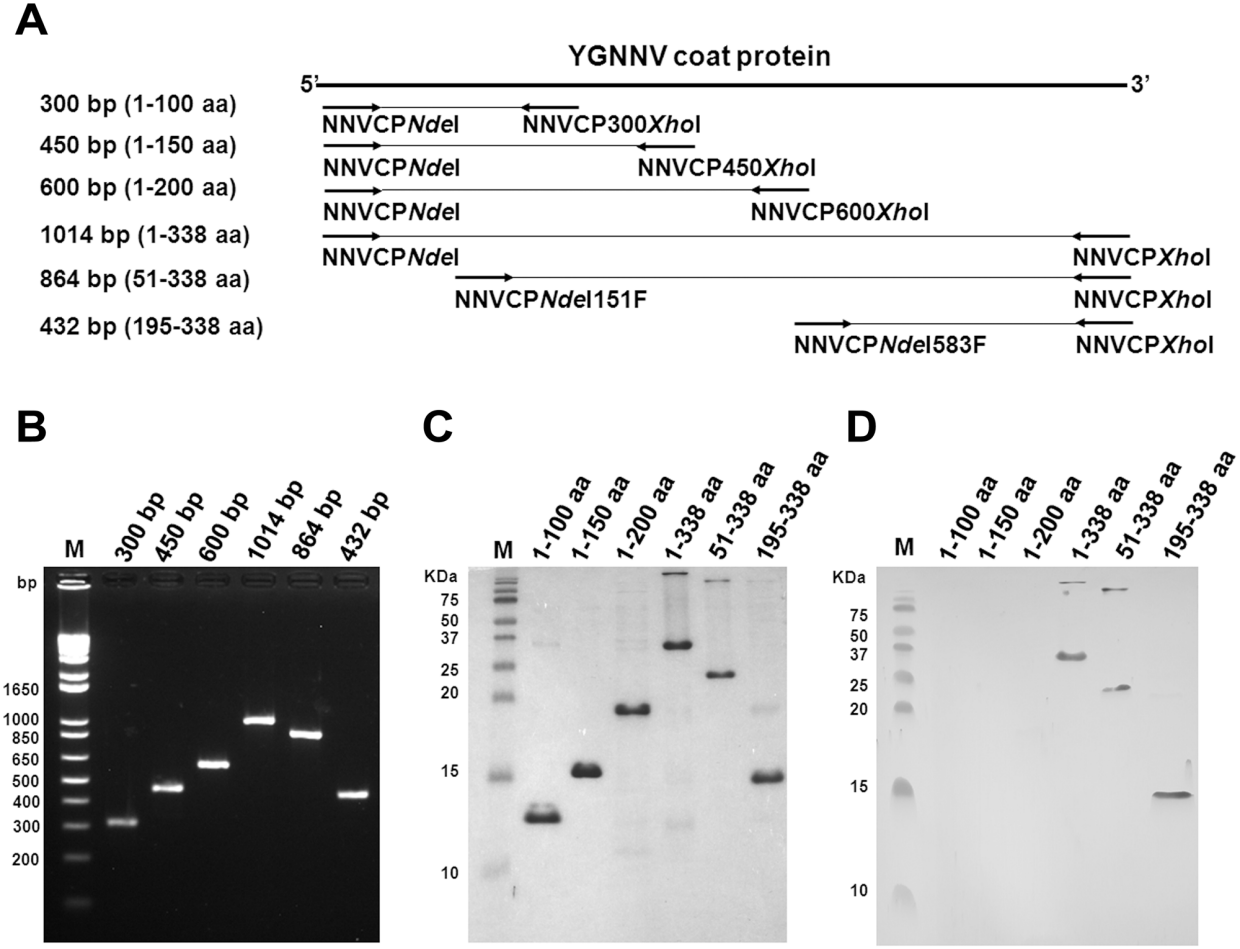
Epitope mapping using serial recombinant YGNNV coat proteins and RG-M18 mAB. (A) Schematic map of serial deletions of recombinant YGNNV coat protein. Specific forward/reverse primers are labeled under the arrow symbols. (B) Serial deletion PCR products. Lane M: DNA nucleotide base pair marker. (C) SDS-PAGE analysis of recombinant proteins expressed from YGNNV coat protein serial deletion clones. Recombinant protein 1–338 aa is the full-length coat protein. Lane M: protein molecular weight marker. (D) Western blot analysis performed using RG-M18 mAB. Lane M: protein molecular weight marker.

In a previous study [16], mAB RG-M18 was identified as a neutralizing antibody against yellow grouper nervous necrosis virus (YGNNV) of the redspotted grouper nervous necrosis virus (RGNNV) strain [22]. To identify the epitope-recognizing site of RG-M18, the YGNNV coat protein sequences were amplified by PCR to generate several fragments (Fig 1A and 1B), and these fragments were then ligated into the *E*. *coli* expression vector pET-20b(+) plasmid (which contains a sequence encoding His-tag for immobilized metal affinity chromatography purification). The recombinant truncated coat proteins were named according to their relative position in the full-length YGNNV coat protein. The recombinant coat proteins were expressed in the IPTG-induced *E*. *coli* BL-21 DE3 strain, and purified by the Ni-NTA column method (Fig 1C). Western blot analysis was performed using the RG-M18 antibody, which revealed significant signals against 1–338 aa (full-length), 51–338 aa, and 195–338 aa constructs, but not 1–100 aa, 1–150 aa, or 1–200 aa (Fig 1D). These data indicate that the epitope of RG-M18 recognition is located in the 195–338 aa region of the C-terminal YGNNV coat protein.

### Fine mapping of the Betanodavirus coat protein epitope using the Pepscan method

**Fig 2:**
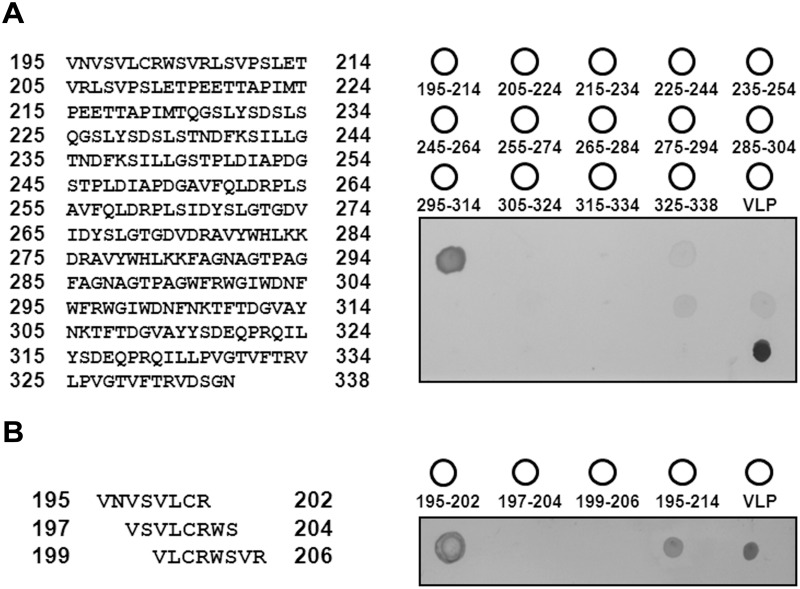
Epitope identification through dot-blotting with synthetic peptides. (A) Left: sequences of the 20-mer synthetic peptides from 195 to 338 aa; each peptide had a 10-mer amino acid overlap with the following peptide. Right: schematic of the peptide array on the PVDF membrane. Virus-like particle (VLP, 1–338 aa) was used as a positive control. Dot-blotting of the 20-mer peptide was performed using RG-M18 mAB. (B) Fine mapping of 8-mer synthetic peptides from 195–206 aa (left). All the assays were performed in triplicate (right).

Subsequently, the 195–338 aa region was further divided into 14 serial sections, each of 20 amino acids (with the exception of the last segment, which consisted of 14 amino acids). The C-terminal sequence of each section overlaps with the N-terminus of the next section by 10 amino acids (Fig 2A). After peptide synthesis, a synthetic peptide array consisting of 13 spots of 20-mer peptides and 1 spot of 14-mer peptide on a PVDF membrane was generated, and subjected to dot blot analysis using RG-M18 mAB. Each synthetic peptide was named according to its location in the coat protein, and the Betanodavirus virus-like particle (VLP, 1–338 aa) was used as a positive control. Peptide 195–214 aa exhibited a strong signal that was similar to the positive control; on the other hand, a signal was not observed between RG-M18 and peptide 205–224 aa, indicating that the peptide 195–204 aa region is the actual site recognized by RG-M18. In addition, a weak signal was observed for the peptide 225–244 aa region; however, similar signals were not observed for peptides 215–234 aa or 235–254 aa, and therefore the weak signal for peptide 225–244 aa may arise from binding at 234 and/or 235 aa. Weak signals were also observed for peptides 275–294 aa and 285–304 aa, indicating that the 285–294 aa region may contribute to the epitope of RG-M18 as well (Fig 2A). The peptide 195–204 aa region was further divided into three serial 8-mer sections, which started at residue 195, 197, and 199, respectively. Dot blot analyses of these three synthetic peptides revealed that only peptide 195–202 aa undergoes a similar interaction with RG-M18 as peptide 195–214 aa and VLP (Fig 2B). Moreover, dot blot analyses of 7-mer peptides 195–201 aa and 196–202 aa showed no binding affinity with RG-M18 (data not shown). Based on these results, the _195_VNVSVLCR_202_ sequence of Betanodavirus coat protein is the epitope site of RG-M18 recognition.

### Alanine-scanning mutagenesis

**Fig 3:**
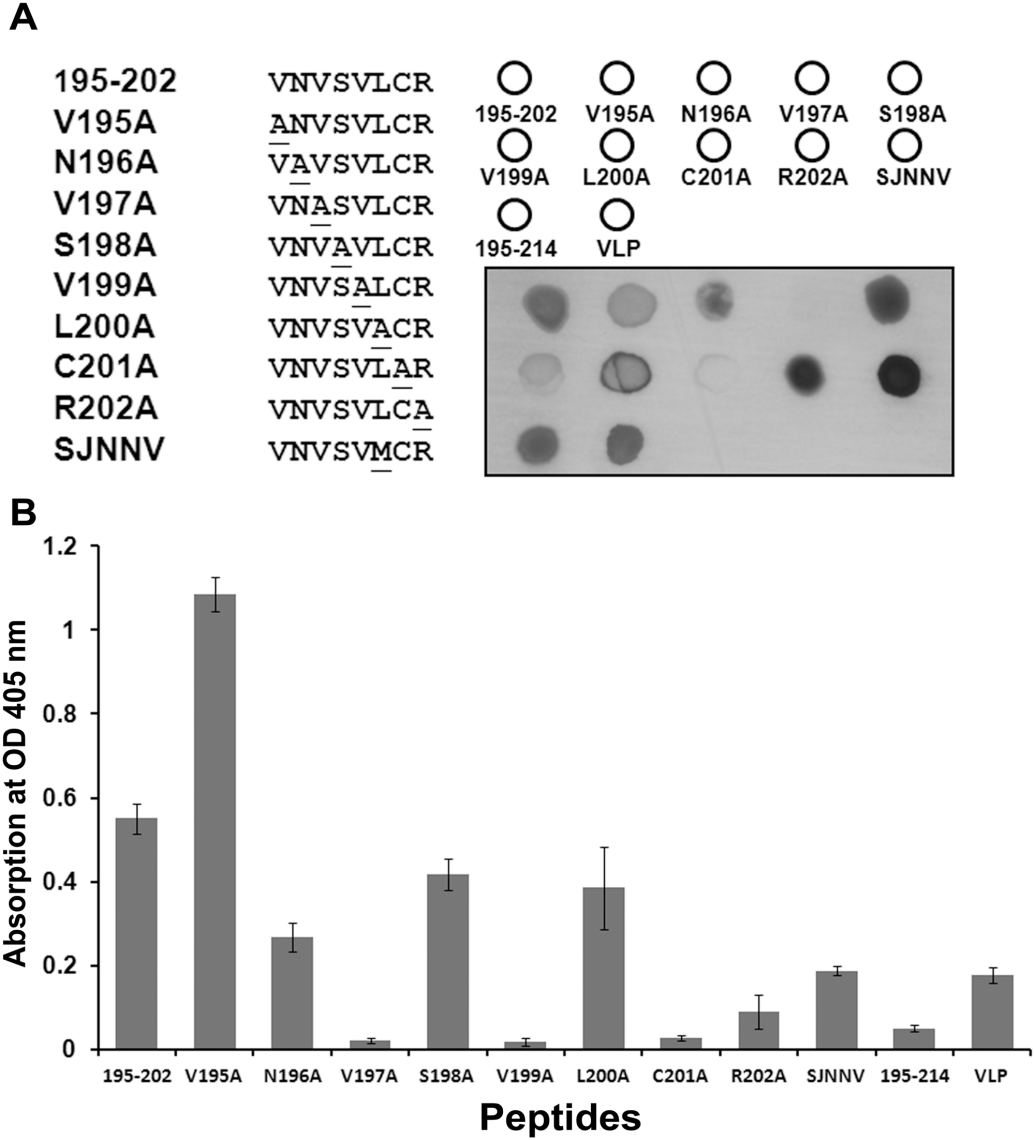
Alanine-scanning mutagenesis of the 195–202 aa epitope. (A) Dot-blotting analysis. Each amino acid of peptide 195–202 aa was individually replaced with Alanine. SJNNV is a peptide in which Leucine 200 is replaced with Methionine. Virus-like particle (VLP, 1–338 aa) and peptides 195–214 aa and 195–202 aa were used as positive controls. The substituted amino acid residues are underlined. This assay was performed in triplicate. (B) ELISA. Each value is the mean ± S.D. (n = 7).

**Fig 4:**
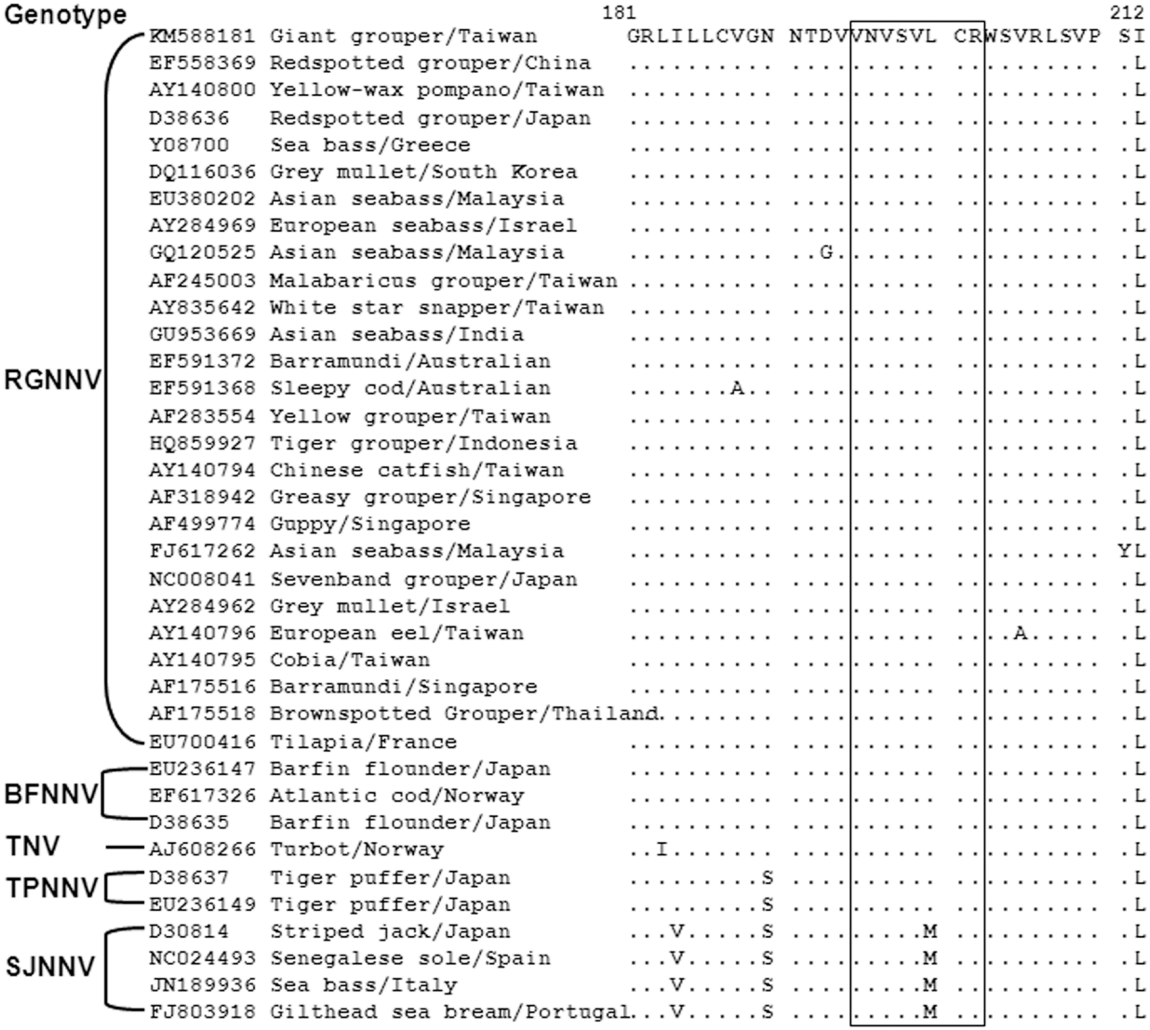
Alignments of coat protein sequence from five Betanodavirus genotypes, covering the regions possessing the epitope recognized by RG-M18 mAB. The boxed sequences indicate the RG-M18-recognizing epitope. The epitopes are conserved, with the exception that residue 200 is Methionine in SJNNV, but Leucine in the sequences of the other four genotypes. A total of 37 isolates were aligned using ClustalX2 and BioEdit 7.2.3 software. GenBank accession numbers are listed in front of the strain name.

The specificity of the interactions between epitope peptide and RG-M18 was further assessed by Alanine-scanning mutagenesis. Constructs were synthesized in which one of the eight residues of _195_VNVSVLCR_202_ was replaced with Alanine. The Alanine mutation peptide array was placed on a PVDF membrane, and peptide 195–202 aa, peptide 195–214 aa, and VLP were used as positive controls (Fig 3A). Dot blotting indicated that the V199A and C201A mutations reduced the binding affinity of RG-M18, while the V197A mutation abolished binding (Fig 3A). The results of ELISA using RG-M18 were similar to those of dot blot analysis. The lowest OD absorption was observed for the V197A, V199A, and C201A mutations (Fig 3B). Comparative sequence alignment demonstrated that all Betanodavirus coat proteins exhibit high conservation at the region of the neutralizing binding site, with the exception that residue 200 is Methionine in SJNNV, but Leucine in the sequences of the other four genotypes (Fig 4). We thus inspected the binding of RG-M18 to peptide sequence _195_VNVSVMCR_202_ (in which the Leucine at position 200 of the SJNNV genotype sequence is replaced with Methionine) by dot blotting and ELISA. To our surprise, RG-M18 recognized _195_VNVSVMCR_202_ as effectively as it recognizes the RGNNV genotype epitope sequence (Figs 3A, 3B and 4).

### Peptide VNVSVLCR protects GB3 cells from GGNNV infection

**Fig 5:**
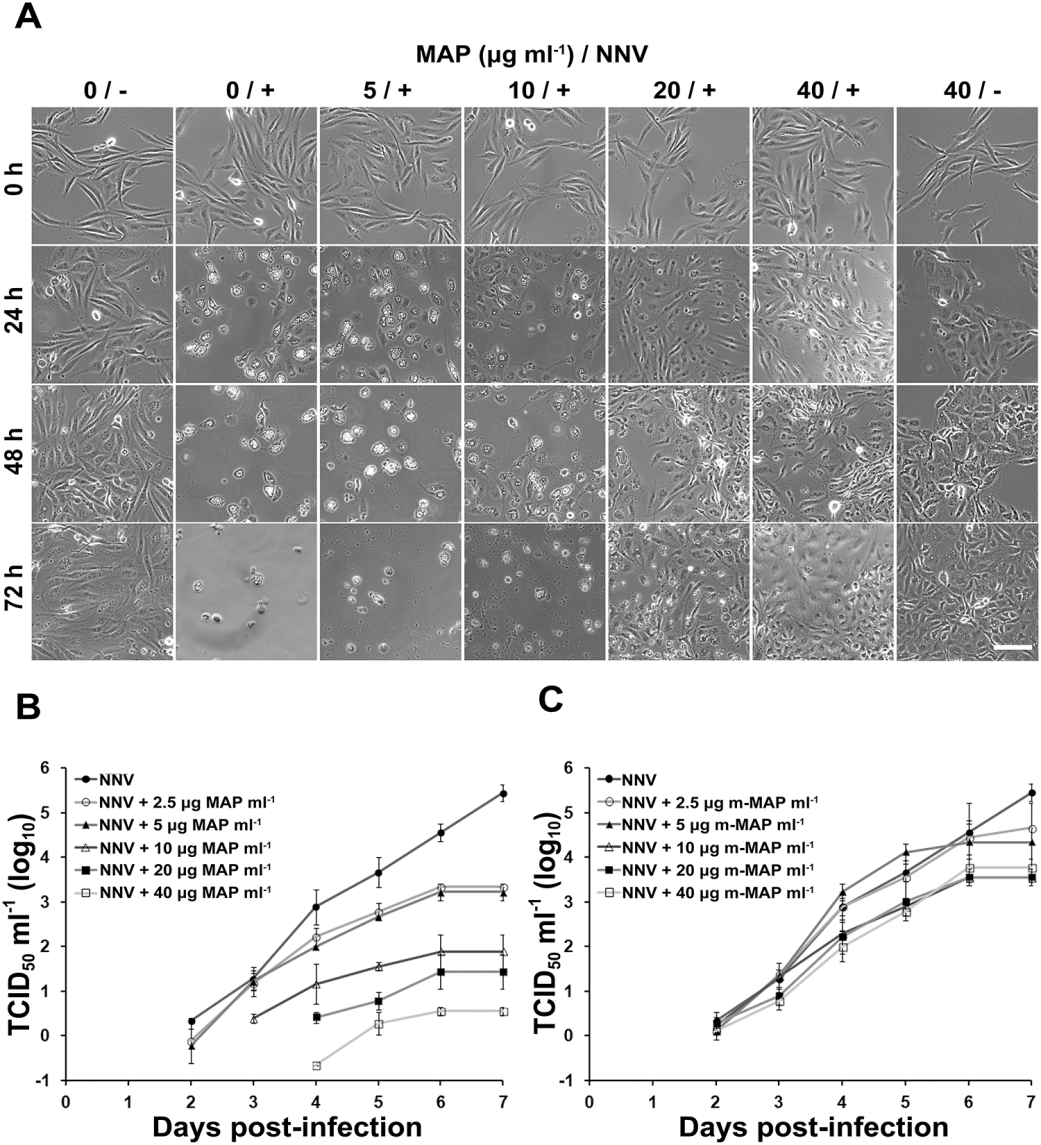
The 8-mer multiple antigen peptide (MAP) protects GB3 cells from GGNNV infection. (A) Microscopic photographs showing GB3 cells infected with GGNNV (m.o.i. = 10) and co-incubated with the indicated concentrations of MAP (0, 5, 10, 20, or 40 μg ml^-1^) at 0, 24, 48, and 72 h. The labels 0/- and 40/- indicate GB3 cells treated with 0 or 40 μg ml^-1^ MAP without GGNNV inoculation, respectively. Labels 0/+, 5/+, 10/+, 20/+, and 40/+ indicate GB3 cells treated with 0, 5, 10, 20, and 40 μg ml^-1^ MAP with GGNNV inoculation, respectively. Scale bar = 100 μm. (B) Protection of GB3 cells from GGNNV infection by co-incubation with the indicated concentrations of MAP (0, 2.5, 5, 10, 20, or 40 μg ml^-1^). (C) Mutant-MAP (m-MAP) offered no protection with the same concentrations of MAP used in (B). Data are shown as means ± S.D. (n = 3).

An 8-branch Lysine scaffold was used to synthesize an 8-mer multiple antigen peptide (MAP) based on the neutralizing epitope sequence, _VNVSVLCR_. GB3 cells were inoculated with GGNNV and co-incubated with different concentrations of MAP. At 24 h post-infection (hpi), typical Betanodavirus-induced cytopathic effect (CPE) was observed in the infected GB3 cells without MAP: infected cells exhibited a round shape, and the cytosol of a single cell contained multiple vacuoles (Fig 5A). Infected cells co-incubated with 5 μg MAP ml^-1^ showed a similar phenomenon to the virus-only control. Although vacuolation was also observed in cells treated with 10 μg MAP ml^-1^, such treatment reduced the numbers of cytopathic cells as compared to the untreated condition. Infected GB3 cells co-incubated with high doses (20 or 40 μg ml^-1^) of MAP did not exhibit Betanodavirus-induced vacuolation. At 48 and 72 hpi, lysis of cells treated with virus alone or co-treated with a low dose (5 or 10 μg ml^-1^) of MAP was observed, whereas CPE was not apparent in cells treated with high doses (20 or 40 μg ml^-1^) of MAP. In addition, the microscopic images indicated that the growth of GB3 cells is not impeded by co-incubation with high doses of MAP (Fig 5A). To better understand the protective effect of MAP, GB3 cells were inoculated with 10-fold serial dilutions of GGNNV (from 10^7^ TCID_50_ ml^-1^) and co-incubated with different concentrations (2.5 to 40 μg ml^-1^) of MAP. Viral CPE was observed continuously for 7 days, and the TCID_50_ titer was calculated every day. A high concentration (40 μg ml^-1^) of MAP effectively protected GB3 cells from GGNNV infection (up to 10^5^ TCID_50_ ml^-1^) (Fig 5B). Co-incubation with the V197A+C201A double mutant MAP (m-MAP) did not result in significant protection (Fig 5C).

### Propagation of GGNNV in GB3 cells was inhibited by co-incubation with MAP

**Fig 6:**
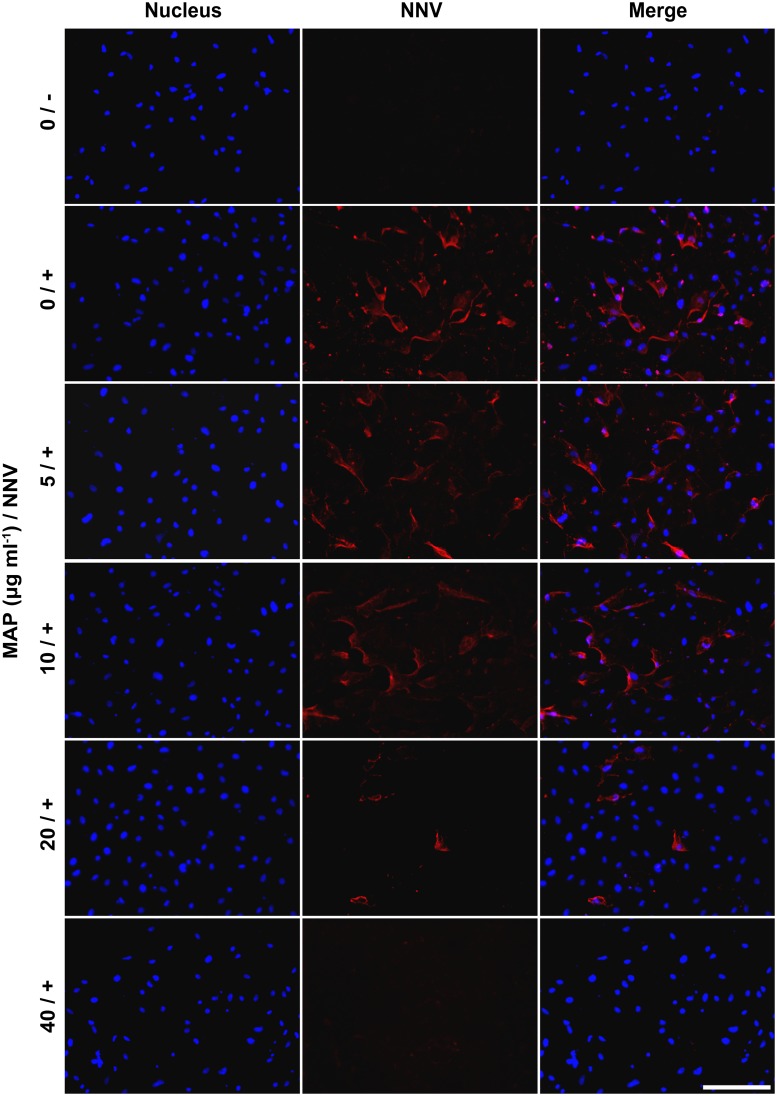
Propagation of GGNNV was inhibited by co-incubation with MAP. Immunocytochemical staining against GGNNV-infected GB3 cells co-incubated with the indicated doses of MAP (0, 5, 10, 20, or 40 μg ml^-1^) was performed at 24 hpi. Label 0/- indicates GB3 cells treated with 0 μg ml^-1^ MAP without GGNNV inoculation. Labels 0/+, 5/+, 10/+, 20/+, and 40/+ are GB3 cells treated with 0, 5, 10, 20, and 40 μg ml^-1^ MAP with GGNNV inoculation, respectively. Betanodavirus viral signal was detected by RG-M18 mAB. Betanodavirus coat proteins were stained with rhodamine (red fluorescence) and cell nuclei were stained with DAPI (blue fluorescence). Scale bar = 100 μm.

GB3 cells were inoculated with GGNNV, and co-incubated with various concentrations of MAP for 24 h. Immunocytochemical staining with RG-M18 revealed Betanodavirus signals in all infected and untreated cells. However, the infection rate was decreased as the MAP dosage increased. Treatment with 20 μg MAP ml^-1^ clearly reduced the density of GGNNV-infected GB3 cells as compared to treatment with 5 or 10 μg MAP ml^-1^. At a dosage of 40 μg MAP ml^-1^, Betanodavirus signals were not detected by immunocytochemical staining at 24 hpi (Fig 6).

**Fig 7:**
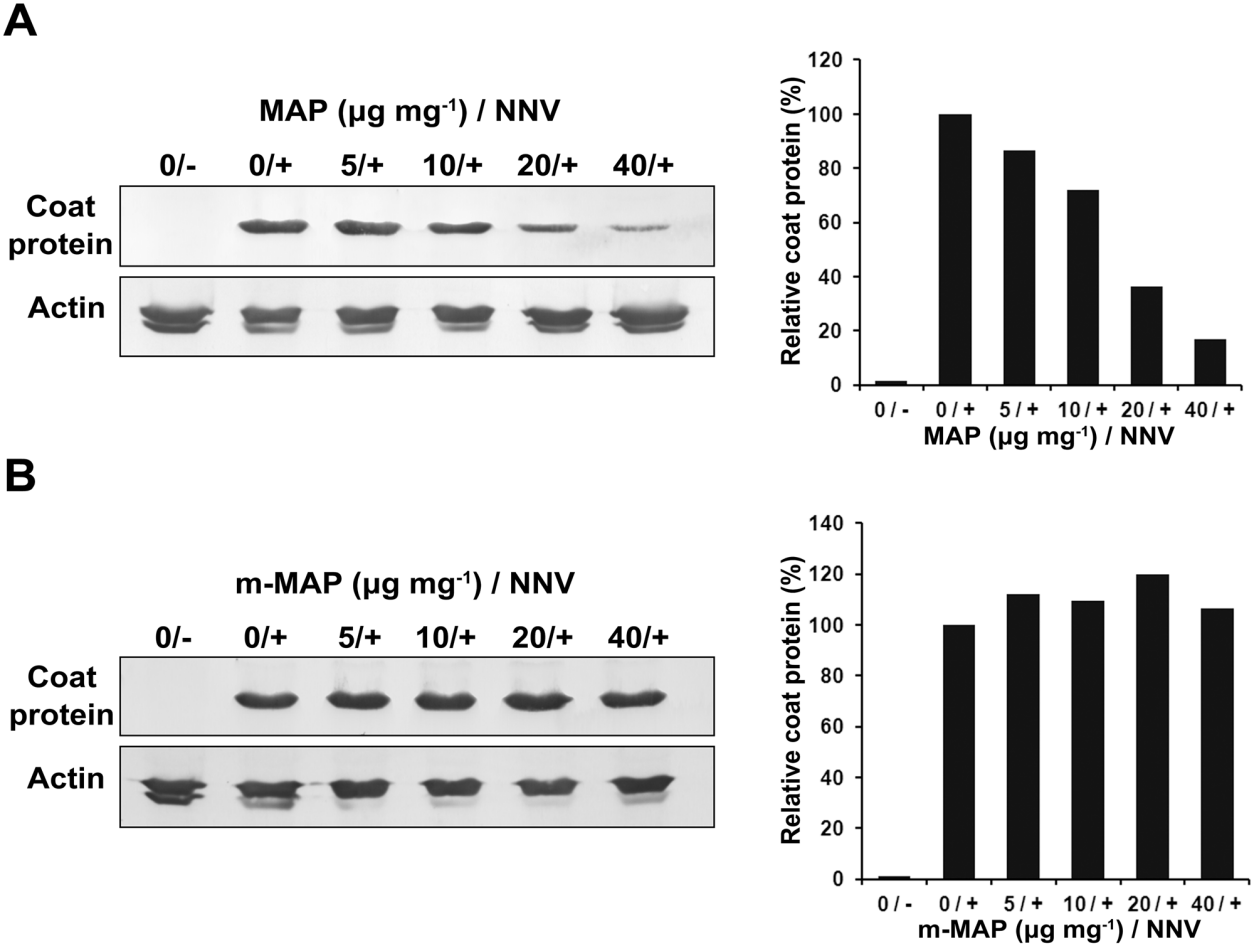
Propagation of GGNNV is inhibited by MAP, but not by mutant-MAP (m-MAP). Western blot and densitometry analyses of GGNNV coat protein in infected cells treated with MAP (A) and m-MAP (B). Total protein was extracted from GGNNV-infected GB3 cells co-incubated with the indicated doses of MAP or m-MAP (0, 5, 10, 20, or 40 μg ml^-1^) at 24 hpi. Label 0/- indicates GB3 cells treated with 0 μg ml^-1^ MAP without GGNNV inoculation. Labels 0/+, 5/+, 10/+, 20/+, and 40/+ are GB3 cells treated with 0, 5, 10, 20, and 40 μg ml^-1^ MAP or m-MAP with GGNNV inoculation, respectively. Betanodavirus coat protein signal was detected using RG-M18 mAB. Actin served as an internal control.

We confirmed the above findings by detecting Betanodavirus coat protein in total protein extracted from infected GB3 cells by Western blot. The quantity of Betanodavirus coat protein was found to decrease as the dosage of MAP was increased. Betanodavirus coat protein quantity in cells co-incubated with 20 and 40 μg MAP ml^-1^ were reduced to 36.10 and 16.87% that in un-treated GGNNV-infected GB3 cells, respectively (Fig 7A). In contrast to MAP, m-MAP treatment did not decrease GGNNV propagation in GB3 cells (Fig 7B).

## Discussion

The aim of this study was to identify the epitope of Betanodavirus coat protein that is recognized by the high-titer neutralizing RG-M18 mAB. Through the use of Western- and dot-blotting analyses, we defined the linear epitope “VNVSVLCR” at position 195–202 near the C-terminal region of the YGNNV coat protein. The epitope has extremely hydrophobic features, with several hydrophobic residues, including three Valines, one Leucine, and one Cysteine (reduced form). The binding ability of each amino acid residue in the epitope was determined by Alanine-scanning mutagenesis combined with dot-blotting and ELISA, revealing that three residues are critical for binding. Of these residues, the amino acids at positions 197 and 199 are the hydrophobic amino acid Valine, while that at position 201 is Cysteine, which, by forming a disulfide bond with a second Cysteine residue, maintains the native fold structure of proteins. Previously, residue Cysteine 201 was shown to be essential for capsid formation of the DGNNV icosahedron structure during *de novo* assembly and reassembly pathways, as well as for the thermal stability of pre-fabricated particles [25]. It is also worth noting that RG-M18 weakly recognized a sequence around aa 234 and 235 in “_SDSLSTNDFK_” and aa 285–294 in “_FAGNAGTPAG_”. However, these sequences have no similarity with “_VNVSVLCR_”, which was strongly recognized by RG-M18. Thus, RG-M18 may recognize multiple epitopes within Betanodavirus coat protein, at varying binding affinities.

Although host specificity differs between Betanodavirus genotypes and their hosts, the neutralizing mAB may recognize epitopes within the same region of the coat protein. The regions corresponding to RG-M18 recognized-epitopes of different viruses from five Betanodavirus genotypes are highly conserved; the sole exception is that the amino acid at position 200 is Methionine in SJNNV, but Leucine in other genotypes (Fig 4). Substitution of Leucine 200 with Methionine in SJNNV caused its binding signal to be the same as that of Betanodaviruses of other genotypes (Fig 3A and 3B). Consequently, RG-M18 mAB can neutralize most Betanodavirus coat proteins. Further investigation is required to determine whether the residue at position 200 imparts the virus-host specificity of infection, but this position is outside of the host specificity determinant region (T4 variable region), identified using chimeric reassortants of SJNNV and RGNNV [15]. Interestingly, the RG-M18 recognizing-epitope (aa 195–202) is located within the neutralization domain (aa 181–212), determined by nine serum samples of naturally-infected sea bass and four mABs [21]. Most of these antibodies recognized multiple regions of 12-mer peptides in the pepscan procedure, with the exception of mAB 3B10, which recognized the sole binding region of 191–202, and exhibited a similar binding region as RG-M18. We should note that a second mAB, RG-M56 [16], also recognized the same epitope (_195_VNVSVLCR_202_) of RG-M18 in our experiments (data no shown). The aa 181–212 epitope region shows high conservation in the coat protein sequences of Betanodavirus of five genotypes (Fig 4); this region is strongly hydrophobic, with two stretches of residues in a strand conformation (183–187 and 195–208) [21]. In a previous study, Nishizawa et al. [20] performed Western blot and matrix plot analysis of the T2 (aa 54–331) and T4 (aa 204–331) regions of the coat protein of SJNNV and Betanodaviruses of other genotypes, allowing them to deduce the existence of a linear epitope, “PAN”, at aa 254–256; PAN is a potential neutralizing epitope for two mABs, 102B and 204D. Although the epitope of RG-M18 appears to be located within a 195–338 amino acid region of the C-terminus of YGNNV coat protein (a region which encompasses the entire region of the T4 protein), RG-M18 did not recognize the 254–256 coresponding sequence of RGNNV coat protein, “PDG”. Interestingly, Nishizawa et al. (1997) also identified a second candidate epitope motif, “AGT”, which appears at both the N-terminus and the C-terminus of the T2 region, which we found weakly binds RG-M18 mAB (in the context of _FAGNAGTPAG_).

Although little research has been conducted on the mechanisms of neutralizing mABs against viral infections, the relationship between neutralizing antibody binding epitopes and viral receptor-binding domain is intriguing. In our competition inhibition test, the multiple-form (MAP) epitope peptide exhibited high ability to protect host cells against GGNNV infection (Fig 6) and inhibit GGNNV propagation (Fig 7). The competitive inhibition of viral propagation by epitope peptide supports the argument that the neutralizing epitope identified by RG-M18 overlaps with the motif required for the attachment of Betanodavirus coat protein to the host cell receptor, a critical early event in infection required for viral entry. The extreme hydrophobicity of the epitope region (as mentioned above) makes it difficult to dissolve the synthetic peptide (especially single peptide aa 195–202) in solution, thus affecting its clinical application. Although the 8-mer multiple antigen peptide with an 8-branch Lysine scaffold exhibits increased dissolution, 20 to 40 μg MAP ml^-1^ are required for the inhibition of an m.o.i. = 10 viral infection. Pharmarceutical modifications may be required to enhance the efficiency of peptides agaist Betanodavirus infection.

In conclusion, we describe here that a novel B-cell linear epitope, VNVSVLCR, of Betanodavirus coat protein is recognized by the neutralizing mAB RG-M18. Amino acids V_197_, V_199_, and C_201_ are the crucial residues for the binding reaction. The competition ability of the neutralizing epitope against GGNNV infection indicates that the epitope identified on the surface of coat protein may contribute to the receptor-binding domain required for the attachment of virus to the host cell. This study thus provides a new insight into the protective mechanism of the neutralizing mAB, and has implications for Betanodavirus vaccinology, as well as peptide drug development.
